# Arctic copepod copper sensitivity and comparison with Antarctic and temperate copepods

**DOI:** 10.1007/s10646-024-02796-2

**Published:** 2024-08-28

**Authors:** Jan Heuschele, Khuong V. Dinh, Torben Lode, Tjalling Jager, Katrine Borgå

**Affiliations:** 1 Department of Biosciences, University of Oslo, P.O Box 1066, Blindern, 0316 Oslo, Norway; 2 DEBtox Research, Stevensweert, The Netherlands

**Keywords:** metal, GUTS, The Nansen Legacy, Calanus, Survival tests

## Abstract

The ongoing global climate crisis increases temperatures in polar regions faster and with greater magnitude than elsewhere. The decline of Arctic sea ice opens up new passages, eventually leading to higher anthropogenic activities such as shipping, fishing, and mining. Climate change and anthropogenic activities will increase contaminant transport from temperate to Arctic regions. The shipping industry uses copper as an antifouling coating. Copper is an essential element but becomes toxic at excess concentrations, and its use may inadvertently affect non-target organisms such as copepods. Copper affects copepods by lowering reproductive output, prolonging developmental time, and causing increased mortality. As data on copper sensitivity of polar copepods at low temperatures are rare, we conducted onboard survival experiments with the Arctic region’s most common copepod species (*Calanus finmarchicus*, *C. glacialis*, *C. hyperboreus*). Acute survival tests were done for up to 8 days on individuals in 70 ml bottles at 1 °C with nominal copper concentrations ranging from 3 to 480 μg L^−1^. We used a reduced General Unified Threshold model for Survival (GUTS) to analyse the data, and placed our results in the context of the few published copper sensitivity data of the Antarctic and temperate copepod species at low temperatures. The sensitivity of Cu exposure was similar between the three *Calanus* species. However, a model comparison suggests that the tested *C. glacialis* population is less sensitive than the other two species in our experiments. Compared to published data, the three Arctic species appear slightly less sensitive to copper compared to their Antarctic counterparts but more compared to their temperate ones. Our literature search revealed only a few available studies on the copper sensitivity of polar copepods. In the future, this species group will be exposed to more pollutants, which warrants more studies to predict potential risks, especially given possible interactions with environmental factors.

## Introduction

The current global climate crisis causes a steady increase in global mean temperatures, with the fastest increase and greatest amplitude in polar regions. The warming has already led to the rapid and massive melting of the inland ice sheet and glaciers, as well as permafrost thawing (Masson-Delmotte et al., 2021), resulting in increased freshwater runoff to the ocean (Haine et al. 2015). Even more striking is the decline of the extent (Comiso 2002; Kwok 2018) and thickness (Kwok and Rothrock 2009) of seasonal and multi-year Arctic sea ice (Onarheim et al. 2018).

The retreating Arctic sea ice opens new shipping passages (Smith and Stephenson 2013; Melia et al. 2016), inevitably leading to increased shipping and exploratory activities. For example, the number of recreational cruises and private boats visiting Svalbard has increased almost threefold since the early 2000s (Linking Tourism & Conservation 2019). The risk of contaminant transport to the Arctic regions will consequently increase (Svavarsson et al. 2021), adding to other long-range transport pathways such as air and ocean currents from temperate regions (Barrie et al. 1992), which are also boosted by climate change (Macdonald et al. 2005; Hung et al. 2022). Higher temperatures and reduced ice cover also allow for more mining, gas, and oil exploitation activities in the Arctic. For example, Norway has recently approved new oil-exploration licenses, and Russia is already drilling for oil in Arctic areas (Buli and Solsvik 2021). Mining activities are also increasing in previously unexploited regions (Haley et al. 2011).

While still underdeveloped in the Arctic, aquaculture activity might increase at higher latitudes when the environmental conditions become more favourable (Froehlich et al. 2018). The combination of shipping, tourism, and resource exploitation will likely lead to higher contaminant exposure throughout the polar region, including coastal and pelagic marine habitats.

Ecotoxicological research in Arctic habitats has so far focused on the consequences of petroleum hydrocarbons (Toxværd et al. 2018b, a, Toxværd et al. (2019)), persistent organic pollutants (Muir and de Wit 2010), and mercury (Kirk et al. 2012). The impact of metals, such as copper, on small invertebrates in the marine environment, has not received much attention. Copper is a trace element essential for many metabolic processes but becomes toxic at excess concentrations, especially for unicellular algae and invertebrates (Brand et al. 1986; Hebel et al. 1997). The shipping and aquaculture industry uses copper as an antifouling coating, which may inadvertently affect non-target organisms such as copepods. These zooplankton species are small but very abundant organisms in the world’s oceans and a crucial link in the marine food web (Humes 1994). Copper affects copepods already at sub-lethal concentrations by reducing reproductive output (Kwok et al. 2009) and prolonging developmental time (Lode et al. 2018). At higher concentrations, copper causes mortality (Sahlmann et al. 2019). However, the copper sensitivity varies within and between species and also depends on other environmental parameters such as temperature (Heuschele et al. 2022).

**Fig. 1:**
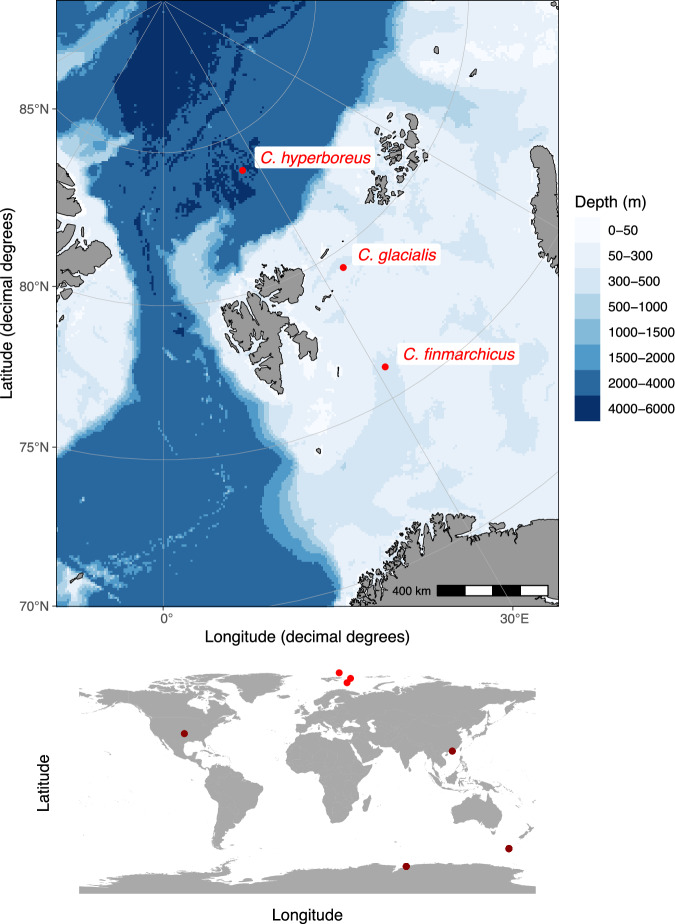
Upper Map: Sampling locations of the animals used in the onboard experiments. Lower Map: The origin of the copepods used in the published low-temperature LC50 experiments

Survival data on copepods exposed to copper at low temperatures are rare. In a recent review, we identified only six studies where species were exposed to copper at temperatures below 10 °C (Heuschele et al. 2022), four of which were from polar regions (see Fig. 1 for a map of species and test locations). In addition to closing the knowledge gap, our study was motivated by the increased risk of Cu exposure posed by climate change. We compared the copper sensitivity of field-caught individuals of the three most abundant copepod species of the Arctic: *Calanus hyperboreus, C. glacialis*, and *C. finmarchicus*, targeting survival in a concentration-response experiment. The three species belong to the genus *Calanus* but differ in maximum size, reproductive strategy and generation time (Table 1), with *C. hyperboreus* being the biggest and *C. finmarchicus* the smallest species. Although their geographic distribution ranges partially overlap, *C. finmarchicus* is considered a more temperate species, while *C. glacialis* and *C. hyperboreus* are considered Arctic species, with their main distribution range being in Subarctic and Arctic Seas (Kosobokova et al. 1997; Kvile et al. 2018).

**Table 1:** Calanoid copepod sampling sites and conditions in the Barents Sea, onboard short-term copper exposure survival experiments and nominal concentrations, x indicates tested copper concentrations

Copper Concentration (µg L^−1^)	*C. finmarchicus*	*C. glacialis*	*C. hyperboreus*
3	x	x	x
16	x	x	x
23	x	x	x
31	x	x	x
44	x	x	x
120		x	x
240	x	x	x
480		x	x
*Experimental information*			
Date (Year-Month-Day)	2021-03-08	2021-03-14	2021-09-02
Cruise ID	Q1	Q1	JC2-2
Sampling depth (m)	306	312	100
Bottom depth (m)	326	332	4002
Latitude (N)	75.9981	79.4500	83.8436
Longitude (E)	31.2192	34.0000	25.0386
Sampling gear	Bongo net 180 µm	Bongo net 180 µm/WP3 1000 µm	Bongo net 180 µm
Life stage	CIV-CV	Adult female	Adult female
Average prosome size (mm)*	2.76 ± 0.32	4.40 ± 0.38	5.25 ± 0.21
Replicates x Concentrations	5 × 6	5 × 8	5 × 8
Exposure duration (d)	4	6	8
Temperature (°C)	0-1	0-1	0-1
Salinity (PSU)	34.9	34.9	34.9

^*^Sizes of *C.finmarchicus* and *C.glacialis* were measured from samples preserved in Lugols solution of the experimental animals and corrected for shrinkage following Jaspers and Carstensen (2009). Sizes of *C. hyperboreus* were measured from images of the same population, taken at the same time as the experiment. Further information about the physiochemical conditions at the sampling stations can be found in Gerland et al. (2022) and Fransson et al. (2022)

The lower surface area-to-volume ratio of big species (Rand 1995) and reduced mass-specific sodium uptake rates (Grosell et al. 2002) are expected to slow down the specific water-borne copper uptake compared to smaller species. Therefore, we expected that in the largest species, *C. hyperboreus*, effects of copper exposure would appear later compared to the smaller *C. glacialis and C. finmarchicus*. However, given the remote locations of the Arctic habitats, *C. hyperboreus* have historically been less exposed to pollutants compared to coastal temperate and tropical species (Halpern et al. 2008).

## Method

To assess the copper sensitivity of three Arctic copepod species, we conducted classic acute exposure experiments, comparable to the OECD guidelines (OECD 2004), with field-caught *C. finmarchicus*, *C. glacialis*, and *C. hyperboreus* in a climate-controlled room (1 °C) onboard the research vessel Kronprins Haakon. The experiments were performed on two research cruises in the spring (*C. finmarchicus, C. glacialis*) and autumn (*C. hyperboreus*) of 2021; Table 1 and Fig. 1 detail the experimental conditions, dates, and sampling sites.

Nominal exposure concentrations (Table 1) were based on the 0.01, 20, 40, 60, 80, and 99.9 percentile of the predicted LC50s for a combined dataset of all available Calanoida copepods studies, assuming a 48-hour exposure, 1 °C, and a salinity of 34 PSU (Heuschele et al. 2022). After the experiments, we discovered that the stock solution molarity was a magnitude more diluted than what we used in the calculation to prepare the test concentrations; hence the used nominal concentrations were 10-fold lower than targeted. Consequently, the lower range of tested exposure concentrations did not cause any mortality in *C. finmarchicus* in our experiments on the first cruise. We included two additional concentrations in the *C. glacialis* and *C. hyperboreus* experiments, representing half and twice the previous highest concentration. For the same reason, we increased the exposure duration from 4 days for *C. finmarchicus*, 6 days for *C. glacialis*, and 8 days for *C. hyperboreus*. We could not test the actual copper concentration in the experiments since it was below the detection limit of the methods available to us. However, calibration curves at higher concentrations showed a linear response between the nominal and measured copper concentrations.

We caught all copepods with a vertical plankton haul from approximately 300 m to the surface for *C. glacialis* and *C. finmarchicus*, and from 100 m for *C. hyperboreus*. Once on board, we placed them for at least 24 h in 1-liter plastic bottles with filtered seawater (0.2 µm) for gut evacuation, with 25 individuals of *C. finmarchicus*, 20 *C. glacialis*, and 15 *C. hyperboreus* per bottle. *C. finmarchicus* were at copepodite stage CIV-CV, while the other species were adult females.

We then filled 70 ml cell culture bottles with 56 ml of 0.2 µm filtered seawater, added 0.002 ml of the respective treatment stock dilution, closed the lid, and rotated thrice to mix the solution. We had enough animals to test five individuals in each concentration (see Table 1). To minimize the transfer of algae and bacteria, the copepods were individually transferred thrice from one small glass beaker filled with 0.1 µm filtered seawater to another using a Pasteur pipette. After this cleaning procedure, we added them to individual cell culture flasks by pipetting them with 2 ml of seawater. The bottles were kept in the dark for the duration of the experiment to mimic the dark winter and diapause period.

We visually checked the state of each individual every 12 h until the end of the respective experiment (Table 1), when we noted the animals’ final state and transferred them individually to Eppendorf tubes with Lugol’s solution for the preservation and later size measurements. The samples for *C. hyperboreus* size measurements were unfortunately lost during transport. Therefore, we measured sizes from other photographed individuals of the same *C. hyperboreus* population and stage, that we sampled during the same cruise.

### Data analysis

To analyse the effect of copper on survival and potential differences between species, we used a reduced General Unified Threshold model for Survival (GUTS) (Jager et al. 2011; Jager and Ashauer 2018). We choose GUTS in addition to classic survival models since it accounts for the toxicokinetic and -dynamics of toxicant effects (Ashauer and Escher 2010). While classic survival models are simpler and more focused on predicting survival probabilities in specific exposure conditions, after a standardized exposure time, GUTS models provide a more mechanistic understanding of stressor effects in ecotoxicological studies. Being able to estimate GUTS parameters describing the uptake and damage dynamics allows us to a) provide a more meaningful comparison between species, b) predict toxicant effects beyond the experimental duration and c) model the effects of exposure scenarios where toxicant concentrations vary over time. The latter could, for example, be used to predict survival under pulsed scenarios.

**Fig. 2:**
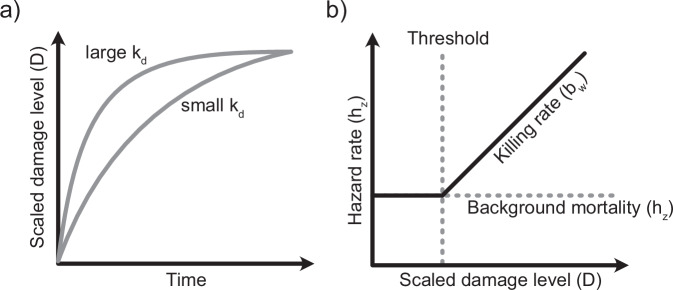
Schematic illustrations of the role of the GUTS parameters. **a** the dominant rate constant kd describes the dynamics of how the external concentration is related to internal damage. **b** The relationship between damage level and hazard rate, including parameters background mortality (h_z_), killing rate b_w_ determining the slope of the relationship, and the median threshold damage level (m_w_), which is referenced to the external contaminant concentration (Figure adapted from Jager and Ashauer 2018)

We used the GUTS package of the Build Your Own Model (BYOM) framework under MATLAB (MATLAB 2021), adapted to allow parameters to differ between simultaneously fitted data sets. For model optimization and construction of confidence intervals, we used the methods described in detail by Jager (2021). We tested models with stochastic death (SD) and individual tolerance (IT) as death mechanisms but focused on the GUTS-SD model results since these models fitted better to our data, whereas the GUTS-IT results are shown in the supplementary information (SI Tables 1, 2). The GUTS-SD involves four model parameters: the dominant rate constant *k*_*d*_, the threshold damage level *m*_*w*_, the killing rate *b*_*w*_, and the background hazard rate *h*_*b*_. Given that all copepods survived in the controls, we fixed the background mortality *h*_*b*_ in all models to a low default value of 0.001 d^−1^. Fig. 2 explains the role of the different GUTS parameters in the model.

We formulated eight different models, starting with three single-species models, where we fitted all parameters for each species separately (M0, nine free parameters). The sums of the AICs and Minus LogLikelihoods (MLL) were then used as a baseline to compare it with the other models, where we fitted the three species simultaneously. In model M1, we fitted one set of parameters to the pooled data from all three species (three free parameters). Additionally, we formulated three models where we allowed one parameter to be species-specific: the dominant rate constant *k*_*d*_ (Model M2), killing rate *b*_*w*_ (Model M3), the median of the threshold distribution *m*_*w*_ (Model M4). All these models have five free parameters.

Additionally, we considered a model (M5) that assumes that all species are equally sensitive, intrinsically (i.e., the same amount of damage causes the same effect on survival), but differ in the amount of damage they accumulate in a steady state. GUTS models work with scaled damage, and we can use a weight factor *w* to scale the relative damage term (*D*_*w*_) for each species (for one species, *w* is fixed to 1, so we again have five free parameters). This approach was developed for mixtures of chemicals with the same mechanism of action (Bart et al. 2021) but was here adapted to the same chemical in different species. Differences in steady states between the three species could, for example, arise due to species-specific abilities to detoxify copper ions.

The models were evaluated by comparing the goodness of fit using their difference in negative log-likelihood (likelihood-ratio test) and by the Akaike Information Criterion (AIC). The likelihood ratio test, a formal test for significance, can be performed to compare nested models (i.e., one model is derived from the other by fixing one or more parameters to a specific value). For non-nested models, we need to resort to the AIC. The likelihood-ratio test and the AIC both consider how many free parameters are used when fitting the model. Additionally, we provide the R^2^ for the fits, even though this metric is less suitable for discrete data such as our survival counts.

To place our results in context with the published literature, we calculated the LC50s using BYOM’s in-built functionality from the simplest model M1 as the copper sensitivity differences between the three tested species were small.

To compare the GUTS results with a classical survival model, we also ran a Cox proportional hazard model using the Survival package in R (Therneau and Grambsch 2000; Therneau 2021; R Core Team 2022). Due to the unbalanced exposure durations among species, we restricted this to the first four days of data. The model was parameterised using copper concentration and species identity as additive explanatory terms. We did not include an interaction between the explanatory terms, as it led to convergence issues and gave unreliable estimates.

### Literature data

We extracted studies at temperatures up to 10 °C using the source data from a meta-analysis on copper toxicity drivers in copepods (Heuschele et al. 2022). These were omitted from the original meta-analysis (Heuschele et al. 2022) due to collinearity between longer exposure times and experimental temperatures, where short experiments are done mostly at high temperatures and experiments done at low temperatures have long exposures. This left 47 LC50 entries from 6 articles (Boeckman and Bidwell 2006; Bao et al. 2008; Li et al. 2014; Marcus Zamora et al. 2015; Holan et al. 2016, 2019) for copepods at experiments below 10 °C (SI Table 3, Fig. 4).

To test which factors might influence copper sensitivity in copepods, we ran linear mixed-effect models on the combined dataset of our experimental results and the literature values. We formulated four separate models because some environmental and experimental factors correlated in our limited dataset. The models included either latitude, temperature, taxonomic order, or body length as explanatory terms, with the reported LC50 as the dependent variable. The values for LC50 and exposure duration were log-transformed before analysis to avoid skewed data distributions. We included species and exposure duration as a random effect in each model to account for repeated measurements of the same species, except for the model for exposure duration itself. For the model with latitude, we also included a fixed factor indicating the hemisphere and allowed for an interaction with latitude in the starting model. The mixed-effects models were run using the lme4 package (Bates et al. 2015) in R version 4.2.0 (R Core Team 2022). Each model’s marginal and conditional R^2^ was calculated using the function provided by the MuMIn package (Bartoń 2023). We used a Chi-square test to compare each model with a null model that only included the random factors.

## Results

**Fig. 3:**
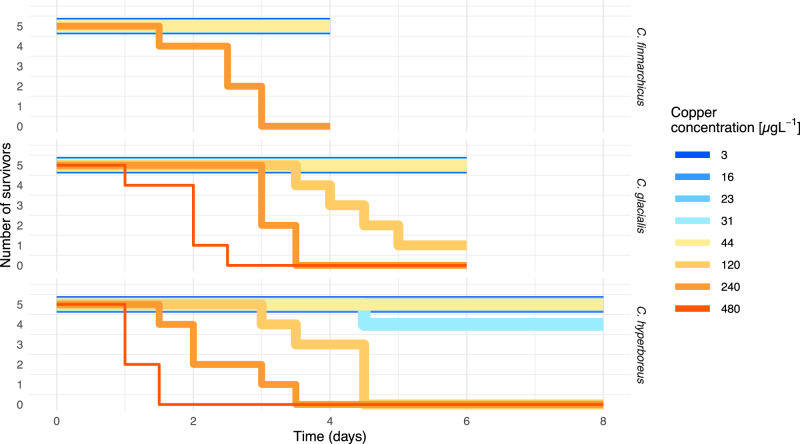
Survival data from the three experiments. The length of the exposure duration differed between the experiments 4, 6, 8 days), since we needed to adjust it to control for the low mortality in the experiments. Line thickness decreases with increasing copper concentration

The copper sensitivity of the three *Calanus* species was similar, judging from the survival patterns over time. All models with stochastic death (SD) as the mortality mechanism had better AIC values compared to the individual threshold (IT) models (SD results in Table 2, IT results in SI Table 1, 2). Depending on the species, the model where all species were fitted with one set of parameters (M1) already explained between 94 and 98% of the data (Table 2, SI Figs. 1–3), suggesting that the three species respond very similarly to copper. This conclusion is reflected by the raw data patterns (Fig. 3). Based on MLL, M1 is not significantly worse than M0. The MLL of M1 is higher (reflecting a poorer fit), but this is counteracted by the fact that it has six parameters fewer. Models M2-M5 are also nested into model M0 and are not statistically different from M0. Model M1 is nested in the models M2-M5. We can see that they are significantly better than M1.

**Table 2:** Model performance comparison

Model	Description	Species	Parameters fitted	AIC	MLL	R^2^	NRMSE
M0	Species specific models -	*C. finmarchicus*	3	22.18	8.09	0.9747	0.0414
		*C. hyperboreus*	3	65.81	29.91	0.9775	0.0872
		*C. glacialis*	3	55.74	24.87	0.9735	0.0733
		Sum	9	143.73	62.87		
M1	No species-specific parameters	*C. finmarchicus*	3	142.11	68.06	0.9779	0.0387
		*C. hyperboreus*				0.9635	0.1111
		*C. glacialis*				0.9385	0.1116
**M2**	**Species- specific** ***k***_***d***_	***C. finmarchicus***	**5**	**137.92**	**63.96**	**0.9734**	**0.0425**
		***C. hyperboreus***				**0.9754**	**0.0913**
		***C. glacialis***				**0.9618**	**0.0880**
**M3**	**Species- specific** ***b***_***w***_	***C. finmarchicus***	**5**	**138.22**	**64.11**	**0.9760**	**0.0404**
		***C. hyperboreus***				**0.9763**	**0.0895**
		***C. glacialis***				**0.9653**	**0.0838**
**M4**	**Species-specific** ***m***_***w***_	***C. finmarchicus***	**5**	**140.03**	**65.01**	**0.9732**	**0.0427**
		***C. hyperboreus***				**0.9734**	**0.0948**
		***C. glacialis***				**0.9541**	**0.0964**
M5	Species specific w	*C. finmarchicus*	5	138.50	64.25	0.9732	0.0427
		*C. hyperboreus*				0.9751	0.0917
		*C. glacialis*				0.9579	0.0923

*AIC* Akaike information criterion, *MLL* Minus log-likelihood of each model. R^2^ and *NRMSE* Normalized Root Mean Square Error for each dataset are calculated on means, including t = 0, from the residuals. The competing best models based on AIC are in bold font

The better fit of the models that allow for species-specific parameters is also confirmed when comparing all models using AIC. We found that the three competing best models M2, M3, and M5 are within ΔAIC < 2 (Table 2), and thus have equal support. In all these models, *C. glacialis* was more sensitive than the other two species. M2 suggests that *C. glacialis* had a lower dominant rate constant *k*_*d*_ than the other two species. M3 indicated that the killing rate (*b*_*w*_) of *C. glacialis* was around 60% and 100% lower than for *C. finmarchicus* and *C. hyperboreus*, respectively. Similarly, the relative damage term *w* in M5 was 21 and 28% higher in *C. finmarchicus* and *C. hyperboreus*, than *C. glacialis* (Table 3). In any case, the adequate performance of model M1 based on all species and the overlapping confidence intervals of the parameter estimates shows that the differences between the species are minor.

**Table 3:** Parameter estimates with lower and higher 95% confidence limits (CI) of all compared models

Model	Parameter	Species	Estimate	lower CI	higher CI
M0	*k*_*d*_ (d^−1^)	*C. finmarchicus*	0.001641	0.001641*	6.003
	*b*_*w*_ (L µg ^−1^ d^−1^)	*C. finmarchicus*	2.737	0.004260	8.728
	*m*_*w*_ (mol L^−1^)	*C. finmarchicus*	0.3913	0.1462	237.6*
	*k*_*d*_ (d^−1^)	*C. hyperboreus*	0.2225	0.01994	0.6001
	*b*_*w*_ (L µg ^−1^ d^−1^)	*C. hyperboreus*	0.04698	0.01493	0.4885
	*m*_*w*_ (µg L^−1^)	*C. hyperboreus*	48.36	5.813	87.54
	*k*_*d*_ (d^−1^)	*C. glacialis*	0.001641	0.001641*	0.2804
	*b*_*w*_ (L µg ^−1^ d^−1^)	*C. glacialis*	2.254	0.01272	4.228
	*m*_*w*_ (µg L^−1^)	*C. glacialis*	0.5552	0.3560	62.99
M1	*k*_*d*_ (d^−1^)	*Calanus sp*.	0.1194	0.001641*	0.3764
	*b*_*w*_ (L µg ^−1^ d^−1^)	*Calanus sp*.	0.03986	0.01372	2.844
	*m*_*w*_ (µg L^−1^)	*Calanus sp*.	28.14	0.4492	69.07
M2	*k*_*d*_ (d^−1^)	*C. finmarchicus*	0.1155	0.01777	0.3286
	*k*_*d*_ (d^−1^)	*C. hyperboreus*	0.1280	0.01766	0.3763
	*k*_*d*_ (d^−1^)	*C. glacialis*	0.08974	0.01366	0.2509
	*b*_*w*_ (L µg ^−1^ d^−1^)	*Calanus sp*.	0.05333	0.01900	0.3511
	*m*_*w*_ (µg L^−1^)	*Calanus sp*.	27.80	4.793	61.73
M3	*k*_*d*_ (d^−1^)	*Calanus sp*.	0.1138	0.006278	0.3538
	*b*_*w*_ (L µg ^−1^ d^−1^)	*C. finmarchicus*	0.05584	0.01155	1.083
	*b*_*w*_ (L µg ^−1^ d^−1^)	*C. hyperboreus*	0.08642	0.02559	1.426
	*b*_*w*_ (L µg ^−1^ d^−1^)	*C. glacialis*	0.02890	0.009094	0.4909
	*m*_*w*_ (µg L^−1^)	*Calanus sp*.	28.57	1.868	68.81
M4	*k*_*d*_ (d^−1^)	*Calanus sp*.	0.1163	0.005696	0.2909
	*b*_*w*_ (L µg ^−1^ d^−1^)	*Calanus sp*.	0.04895	0.01851	0.9067
	*m*_*w*_ (µg L^−1^)	*C. finmarchicus*	26.89	1.414	64.46
	*m*_*w*_ (µg L^−1^)	*C. hyperboreus*	25.64	1.549	53.37
	*m*_*w*_ (µg L^−1^)	*C. glacialis*	34.99	2.030	68.17
M5	*k*_*d*_ (d^−1^)	*Calanus sp*.	0.1144	0.01036	0.2913
	*b*_*w*_ (L µg^−1^ d^−1^)	*Calanus sp*.	0.05430	0.01862	0.5951
	*m*_*w*_ (µg L^−1^)	*Calanus sp*.	27.63	2.777	63.12
	*w* (d.l.)	*C. finmarchicus*	1		
	*w* (d.l.)	*C. hyperboreus*	1.077	0.7537	1.080
	*w* (d.l.)	*C. glacialis*	0.8075	0.5695	1.080

*Indicates that the edge of the CI has run into a boundary condition. kd is the dominant rate constant, bw the killing rate, mw the median of the threshold distribution, and w the relative damage term

### Survival model

The classical statistical survival analysis showed that the hazard ratio followed the same relative pattern as the GUTS models, with *C. finmarchicus* and *C. hyperboreus* being similar, while *C. glacialis* has a 4 to 5 times lower risk than the other species (SI Table 4).

### LC50 comparison

**Fig. 4:**
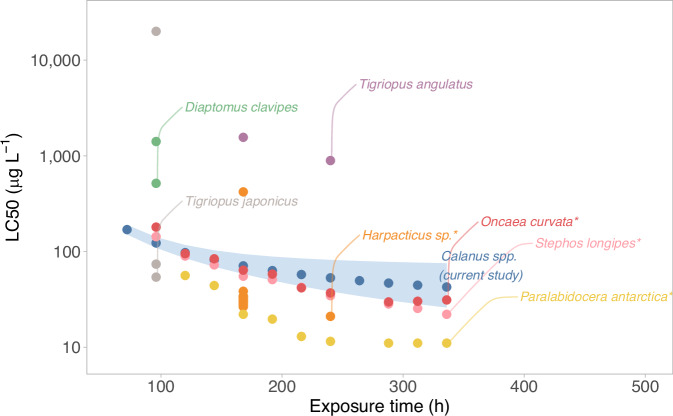
Estimated LC50s from this study in the context of published LC50s of copepods done at low temperatures based on the combined dataset and model M1. The data and references can be found in Table S1. The blue shaded area indicates the range between the low and high 95% confidence interval. *Indicates Antarctic and subantarctic species

The three *Calanus* species from the Arctic appear less sensitive to copper compared to the published LC50 data from low-temperature experiments, either with polar or temperate copepod species (Fig. 4). As expected, increased exposure duration reduced the reported calanoid copepod copper LC50 values. The published LC50s from low-temperature experiments had no clear relation to latitude, but species from the southern hemisphere seem more sensitive compared to the northern hemisphere (Table 4, SI Fig. 4). Given our data, there was no clear relationship between body size, taxonomic order, or temperature with the LC50 (Table 4, SI Fig. 4). Species differences accounted for most of the variation in the data, since all models included species as random factor and all models had high marginal R^2^s (Table 4). Complete model summaries for all relationships are available in the supplementary information (SI Table 5).

**Table 4:** Model summaries for the different drivers of LC50, their marginal (R^2^m) and conditional (R^2^c) R-square values, and the *p* values of the respective Chi-square test against the null model. Estimates and standard errors (SE) are on a log-scale

Model	Variable	Estimate	SE	*t-* value	R^2^m	R^2^c	*p*-value
Exposure duration	Intercept	13.9734	1.9228	7.267	0.341	0.514	<0.001
	log duration (h)	−1.9102	0.3758	−5.083			
Latitude	Intercept	13.2934	1.9357	6.8673	0.485	0.549	0.023
	NorS (South)	−1.053	0.4686	−2.2471			
	log duration (h)	−1.6392	0.4035	−4.0623			
Temperature	Intercept	13.7877	2.0622	6.6859	0.308	0.530	0.894
	Temperature	−0.0064	0.0624	−0.103			
	log duration (h)	−1.8668	0.3937	−4.7417			
Order	Intercept	13.2653	2.0242	6.5533	0.266	0.578	0.933
	Order Cyclopoida	0.0737	0.8995	0.0819			
	Order Harpacticoida	0.1035	0.7212	0.1435			
	log duration (h)	−1.7699	0.3925	−4.5089			
Adult size	Intercept	13.7586	1.9807	6.9465	0.320	0.525	0.885
	adultsize	0.0155	0.1431	0.1084			
	log duration (h)	−1.8708	0.3817	−4.9014			

## Discussion

Despite the large size difference between the three *Calanus* species, we do not find pronounced differences in copper-induced mortality at late copepodite stages, concerning body size or the, in our case, similarly ordered sampling latitude. Although the tested species responded very similarly to copper when we estimated GUTS model parameters separately for each species, the model selection based on AIC suggested lower effects of copper in the medium-sized *C. glacialis* compared to the other two species, with either a lower dominant rate constant *kd*, killing rate *bw*, or lower scaled damage term *w*. However, as the confidence intervals of the parameters overlapped in all models, we cannot be certain which parameters really differ between the three species. Similarly, the survival model indicated an approximately four to five times lower hazard ratio of *C. glacialis* than the other species.

Our results currently represent snapshots of the copper sensitivities in the tested populations, reflecting a combination of environmental effects and genetic differences between species or populations. To disentangle these effects, one needs to repeat the experiment with animals sampling along transects from temperate to polar regions or use longer common garden experiments. These experiments should ideally be done on genera with a large geographic distribution that covers several latitudes, for example, copepods from the genera *Oithona*, *Calanus*, *Pseudodiaptomus*, or *Pseudocalanus*.

Although this study cannot provide a conclusive answer, it is worth discussing the various insights and patterns that have been observed. The potentially lower sensitivity to Cu exposure in *C. glacialis* stands out because it should be less adapted from prior copper exposure than Arctic species and theoretically be more sensitive due to its smaller size than *C. hyperboreus*. The similarity in mortality between the species could be explained by opposing directions of body size- and adaptation-driven sensitivity. Water-borne copper is adsorbed to the carapace of zooplankton and absorbed into the body (Rainbow 1997; Chang and Reinfelder 2002; Barka et al. 2009). Due to the larger surface area, more copper will be adsorbed and absorbed by bigger animals. However, the bigger surface-to-volume ratio of smaller species should result in a faster increase in body-specific copper concentration. We hypothesised that the smaller size of *C. finmarchicus* should lead to an earlier onset of adverse effects of copper exposure in this species due to the higher uptake rates. In contrast, *C. hyperboreus* should be protected by its larger size and slower uptake in this short-term experiment. Arctic species are generally also capital breeders with a high lipid build-up during summer (Falk-Petersen et al. 2009), which could serve as an energy reserve to buffer the costs of coping with stressors. Thus, they should have an advantage over temperate-income breeders with fewer lipids.

While the hypothesised relationship between body size and specific absorption is intuitive, several mesocosm experiments with pesticides based on organic compounds found an inverse relationship with smaller species being less vulnerable than bigger species (Havens and Hanazato 1993). Smaller crustaceans have a higher mass-specific metabolic rate than bigger ones (Makarieva et al. 2008), which could protect them from the effects of excess copper since a higher mass-specific metabolic rate would likely increase the ability of an organism to detoxify and eliminate xenobiotics. These processes might compensate for the hypothesised faster specific absorption and uptake of copper with higher temperatures and thus result in similar copper responses of the tested species. However, the uptake and elimination of insecticides can scale at different rates with temperature (see Mangold-Döring et al. 2022), the ratio of which will contribute to the net toxicity effect.

Besides body size, local phenotypic or genetic adaptation can also influence a species’ vulnerability to contaminants (Moraïtou-Apostolopoulou and Verriopoulos 1979; Becker et al. 2020). Copepods can sometimes adapt to metal contamination by phenotypic or genetic adaptation. *Acartia clausi* from polluted areas had less copper-induced mortality than those originating from a clean area (Moraïtou-Apostolopoulou 1978), while sublethal endpoints such as egg production and ingestion rate were less affected (Moraïtou-Apostolopoulou and Verriopoulos 1979). Two studies indicate that genetic or plastic adaptation to copper exposure is possible in copepods within short timescales. *Tisbe holuthuriae*, a harpacticoid copepod, acquired higher copper tolerance after just five generations of exposure (Moraïtou-Apostolopoulou et al. 1983) and *Tigriopus japonicus* showed plastic physiological adaptation to copper exposure (Kwok et al. 2009).

*Calanus finmarchicus* has a broad distribution range, including temperate regions in the northern hemisphere, while *C. glacialis* and *C. hyperboreus* are more limited to the Arctic or sub-Arctic region. Temperate populations have likely experienced more varying environmental conditions and higher background concentrations of metals and other anthropogenic pollutants in the last century than more northern populations. This may have led to an increase in the antioxidant capabilities of temperate populations and thus a potential for cross-tolerance to handle stress by reactive oxygen species formation from other stressors. For example, prior exposure to heat stress increased the tolerance of brine shrimp *Artemia salina* to cadmium and zinc (Pestana et al. 2016), and sub-lethal doses of one pesticide can increase resistance to other pesticides in frogs and crustaceans (Brausch and Smith 2009; Hua et al. 2014). Such resistance can even be maternally transferred. In freshwater snails, *Biomphalaria glabrata*, offspring gained some cadmium tolerance when the mothers were exposed to predator cues (Augustyniak et al. 2017).

Whether the remotely located species *C. hyperboreus* was potentially more vulnerable in the present study due to its lower past exposure to copper and anthropogenic pollutants needs to be tested in controlled multi-generational experiments and by investigating the geneflow between (sub-) Arctic and temperate populations. A higher transcriptome activity in *C. hyperboreus* compared to the other species when exposed to polycyclic aromatic hydrocarbons (Yadetie et al. 2022) might indicate that *C. hyperboreus* is responding more strongly to anthropogenic pollutants.

Since our sampling campaign was limited in scope, we cannot rule out that variation in sampling period, developmental stage, or life-history strategies such as reproduction also influenced these subtle sensitivity differences between species. Our experiments are thus a first step in comparing copepod species’ vulnerability to anthropogenic pollutants between temperate and Arctic species and populations. We also exposed the copepods to copper for a relatively short time of up to 8 days. In a lifetime and real-world context, the largest and most northern species, *C. hyperboreus*, might still be more vulnerable than the two smaller species since copepod’s generation time scales with size (Gillooly 2000). Given their longer development time, they can be exposed for a long time until they reach reproduction, which could increase the chance of experiencing the adverse effects of copper.

Comparing our results with other published low-temperature experiments from polar and temperate regions suggests that species from the southern hemisphere are slightly more vulnerable than Arctic species. On each hemisphere, we find a potential latitudinal gradient of decreasing copper LC50s towards the poles in copepods, potentially explained by the different past and current exposure to pollutants and adaptation to variable environmental conditions. Antarctic organisms evolved in relative isolation for the last million years (Clarke et al. 2007) and might, therefore, be less adapted to cope with pollution in general, for example, by lacking cross-tolerance by an evolved upregulation of anti-oxidant production. Similar findings are reported for other marine and terrestrial invertebrates; e.g. when comparing sea urchins, the effects of copper and cadmium until reaching a specific larval state was higher in the Antarctic species *Sterechinus neumayeri* than in closely related temperate species (King and Riddle 2001). However, it must be noted that our latitudinal comparison included temperate copepod species tested at low temperatures, which were probably outside their optimal thermal range, in contrast to our experimental study, where all species were within their optimal range. The generally low data availability for polar copepods also indicates a general knowledge gap between temperate and polar species.

Due to increased human activity, the risk of adverse effects through copper contamination in the Arctic will probably increase. The availability of free copper ions determines, for the most part, copper toxicity in the water (Grosell and Wood 2012). The ions readily bind to organic substances and particulate matter, which sink out of the water column or get ingested via food uptake. Therefore, the toxicity caused by shipping and aquaculture might stay limited to the vicinity of the sources, such as harbours and net pens. Copper sensitivity often scales with the environmental temperature in many species and taxa (Boeckman and Bidwell 2006; Gomiero and Viarengo 2014; Heuschele et al. 2022). In a hotter climate, copper could also affect seemingly insensitive species, especially considering potential interactions with other pollutants and environmental factors (e.g., pH, predation risk). The concentration of other metals and pollutants in the Arctic will likely increase with anthropogenic activity. Additive or synergistic effects between different contaminants and their interactions with environmental factors are well-documented (Holmstrup et al. 2010; Laskowski et al. 2010), but much less has been studied for the Arctic and Antarctic organisms (Dinh et al. 2022). Exposure in nature can also last longer than in short-term experiments, which could alter effective threshold concentrations. In addition, sublethal effects on reproduction and motility can adversely affect population dynamics (Saaristo et al. 2018); such effects were not considered in our study and would generally occur at lower exposure concentrations. Therefore, humankind must aim to reduce additional contaminant releases in polar areas before pollutants become a major problem in these regions too.

## Supplementary Information

Supplementary Information

Supplementary Information
