# Supplementary material for: Arctic copepod copper sensitivity and comparison with Antarctic and temperate copepods
Source: Ecotoxicology. 2024 Aug 28;33(9):1026–38. doi: 10.1007/s10646-024-02796-2 (PMC11480128; doi:10.1007/s10646-024-02796-2)
Supplement: Supplementary file 1 — Supplementary Information [file 10646_2024_2796_MOESM1_ESM.docx]

# Supplementary information

## Supplement Tables

Table 1 Comparison of GUTS model with IT death mechanisms

| **Model** | **Model description** | **Species** |  | **AIC** | **MLL** | **R^2^** | **NRMSE** |
| --- | --- | --- | --- | --- | --- | --- | --- |
| M0 | Species specific models | *C. finmarchicus* |  | 22.39 | 8.19 | 0.9832 | 0.0337 |
|  | Species specific models | *C. hyperboreus* |  | 67.04 | 30.52 | 0.9765 | 0.0892 |
|  | Species specific models | *C. glacialis* |  | 61.00 | 27.50 | 0.9312 | 0.1180 |
|  |  | **SUM** |  | **150.43** | **66.21** |  |  |
| M1 | no species-specific parameters | *C. finmarchicus* |  | 149.15 | 71.57 | 0.9746 | 0.0415 |
|  |  | *C hyperboreus* |  |  |  | 0.9522 | 0.1271 |
|  |  | *C glacialis* |  |  |  | 0.9262 | 0.1223 |
| M2 | Species specific k_d_ | *C. finmarchicus* |  | 142.84 | 66.42 | 0.9812 | 0.0357 |
|  |  | *C hyperboreus* |  |  |  | 0.9765 | 0.0891 |
|  |  | *C glacialis* |  |  |  | 0.9300 | 0.1191 |
| M3 | Species specific F_s_ | *C. finmarchicus* |  | 152.62 | 71.31 | 0.9833 | 0.0337 |
|  |  | *C hyperboreus* |  |  |  | 0.9527 | 0.1265 |
|  |  | *C glacialis* |  |  |  | 0.9262 | 0.1223 |
| M4 | Species-specific m_w_ | *C. finmarchicus* |  | 142.85 | 66.43 | 0.9812 | 0.0357 |
|  |  | *C hyperboreus* |  |  |  | 0.9765 | 0.0891 |
|  |  | *C glacialis* |  |  |  | 0.9299 | 0.1191 |
| M4 | Species-specific w | *C. finmarchicus* |  | 142.85 | 66.43 | 0.9812 | 0.0357 |
|  |  | *C hyperboreus* |  |  |  | 0.9765 | 0.0891 |
|  |  | *C glacialis* |  |  |  | 0.9299 | 0.1191 |

Table 2 Parameter estimates of GUTS models with IT death mechanism. * indicates that the edge of the CI has run into a boundary condition.

| **Model** | **Parameter** | **Species** | **Estimate** | **lower CI** | **higher CI** |
| --- | --- | --- | --- | --- | --- |
| M1 | k_d_ (d^-1^) | *Calanus* sp. | 0.001642 | 0.001641* | 0.07734 |
|  | m_w_ (mol L^−1^) | *Calanus* sp. | 0.9013 | 0.8047 | 38.31 |
|  | Fs | *Calanus* sp. | 2.023 | 1.690 | 2.605 |
| M2 | k_d_ (d^-1^) | *C. finmarchicus* | 0.001641 | 0.001641 | 0.07936 |
|  | k_d_ (d^-1^) | *C. hyperboreus* | 0.001924 | 0.001423 | 0.09485 |
|  | k_d_ (d^-1^) | *C. glacialis* | 0.001336 | 0.001000* | 0.06315 |
|  | Fs | *Calanus* sp. | 1.800 | 1.536 | 2.264 |
|  | m_w_ (mol L^−1^) | *Calanus* sp. | 0.8922 | 0.6837 | 38.51 |
| M3 | Fs | *C. finmarchicus* | 1.702 | 1.278 | 3.470 |
|  | Fs | *C. hyperboreus* | 2.050 | 1.476 | 3.523 |
|  | Fs | *C. glacialis* | 2.110 | 1.614 | 3.305 |
|  | k_d_ (d^-1^) | *Calanus* sp. | 0.001641 | 0.001641* | 0.08325 |
|  | m_w_ (mol L^−1^) | *Calanus* sp. | 0.8988 | 0.7844 | 40.40 |
| M4 | m_w_ (mol L^−1^) | *C. finmarchicus* | 0.8925 | 0.6836 | 36.44 |
|  | m_w_ (mol L^−1^) | *C. hyperboreus* | 0.7616 | 0.7441 | 30.19 |
|  | m_w_ (mol L^−1^) | *C. glacialis* | 1.095 | 1.065 | 43.35 |
|  | k_d_ (d^-1^) | *Calanus* sp. | 0.001641 | 0.001641* | 0.07136 |
|  | Fs | *Calanus* sp. | 1. 1.800 | 1.536 | 2.264 |
| M5 | w (d.l.) | *C. finmarchicus* | 1 |  |  |
|  | w (d.l.) | *C. hyperboreus* | 1.172 | 0.8668 | 1.559 |
|  | w (d.l.) | *C. glacialis* | 0.8149 | 0.6003 | 1.090 |
|  | k_d_ (d^-1^) | *Calanus* sp. | 0.001641 | 0.001641* | 0.07134 |
|  | m_w_ (mol L^−1^) | *Calanus* sp. | 0.8924 | 0.6835 | 36.45 |
|  | Fs | *Calanus* sp. | 1.800 | 1.536 | 2.264 |

Table 3 LC50 values from published cold-water copper exposure experiments.

| **Species** | **LC50  (ug/L)** | **Exposure  duration (h)** | **Temperature (C)** | **Latitude** | **Total length (mm)** | **Article** |
| --- | --- | --- | --- | --- | --- | --- |
| *Calanus finmarchicus* | 123 | 96 | 0.5 | 75.998 | 3.43 | This study |
| *Calanus glacialis* | 82 | 144 | 0.5 | 79.450 | 5.73 | This study |
| *Calanus hyperboreus* | 63 | 192 | 0.5 | 83.844 | 6.53 | This study |
| *Diaptomus clavipes* | 1411 | 96 | 10 | 35.960 | 1.85 | Boeckman et al 2006 |
| *Diaptomus clavipes* | 516 | 96 | 10 | 35.960 | 1.85 | Boeckman et al 2006 |
| *Harpacticus sp.* | 38 | 168 | 6 | -54.500 | 0.2 | Holan et al. 2019 |
| *Harpacticus sp.* | 31 | 168 | 8 | -54.500 | 0.2 | Holan et al. 2019 |
| *Harpacticus sp.* | 34 | 168 | 10 | -54.500 | 0.2 | Holan et al. 2019 |
| *Harpacticus sp.* | 33 | 168 | 6 | -54.500 | 0.2 | Holan et al. 2019 |
| *Harpacticus sp.* | 26 | 168 | 8 | -54.500 | 0.2 | Holan et al. 2019 |
| *Harpacticus sp.* | 29 | 168 | 10 | -54.500 | 0.2 | Holan et al. 2019 |
| *Harpacticus sp.* | 34 | 168 | 6 | -54.500 | 0.2 | Holan et al. 2019 |
| *Harpacticus sp.* | 28 | 168 | 8 | -54.500 | 0.2 | Holan et al. 2019 |
| *Harpacticus sp.* | 30 | 168 | 10 | -54.500 | 0.2 | Holan et al. 2019 |
| *Harpacticus sp.* | 420 | 168 | 6 | -54.638 | 0.2 | Holan et al. 2016 |
| *Harpacticus sp.* | 21 | 240 | 6 | -54.638 | 0.2 | Holan et al. 2016 |
| *Oncaea curvata* | 180 | 96 | 0 | -68.567 | 0.59 | Marcus Zamora et al. 2015 |
| *Oncaea curvata* | 95 | 120 | 0 | -68.567 | 0.59 | Marcus Zamora et al. 2015 |
| *Oncaea curvata* | 84 | 144 | 0 | -68.567 | 0.59 | Marcus Zamora et al. 2015 |
| *Oncaea curvata* | 64 | 168 | 0 | -68.567 | 0.59 | Marcus Zamora et al. 2015 |
| *Oncaea curvata* | 58 | 192 | 0 | -68.567 | 0.59 | Marcus Zamora et al. 2015 |
| *Oncaea curvata* | 42 | 216 | 0 | -68.567 | 0.59 | Marcus Zamora et al. 2015 |
| *Oncaea curvata* | 37 | 240 | 0 | -68.567 | 0.59 | Marcus Zamora et al. 2015 |
| *Oncaea curvata* | 30 | 288 | 0 | -68.567 | 0.59 | Marcus Zamora et al. 2015 |
| *Oncaea curvata* | 30 | 312 | 0 | -68.567 | 0.59 | Marcus Zamora et al. 2015 |
| *Oncaea curvata* | 31 | 336 | 0 | -68.567 | 0.59 | Marcus Zamora et al. 2015 |
| *Paralabidocera antarctica* | 56 | 120 | 0 | -68.567 | 1.72 | Marcus Zamora et al. 2015 |
| *Paralabidocera antarctica* | 44 | 144 | 0 | -68.567 | 1.72 | Marcus Zamora et al. 2015 |
| *Paralabidocera antarctica* | 22 | 168 | 0 | -68.567 | 1.72 | Marcus Zamora et al. 2015 |
| *Paralabidocera antarctica* | 20 | 192 | 0 | -68.567 | 1.72 | Marcus Zamora et al. 2015 |
| *Paralabidocera antarctica* | 13 | 216 | 0 | -68.567 | 1.72 | Marcus Zamora et al. 2015 |
| *Paralabidocera antarctica* | 12 | 240 | 0 | -68.567 | 1.72 | Marcus Zamora et al. 2015 |
| *Paralabidocera antarctica* | 11 | 288 | 0 | -68.567 | 1.72 | Marcus Zamora et al. 2015 |
| *Paralabidocera antarctica* | 11 | 312 | 0 | -68.567 | 1.72 | Marcus Zamora et al. 2015 |
| *Paralabidocera antarctica* | 11 | 336 | 0 | -68.567 | 1.72 | Marcus Zamora et al. 2015 |
| *Stephos longipes* | 143 | 96 | 0 | -68.567 | 0.81 | Marcus Zamora et al. 2015 |
| *Stephos longipes* | 90 | 120 | 0 | -68.567 | 0.81 | Marcus Zamora et al. 2015 |
| *Stephos longipes* | 72 | 144 | 0 | -68.567 | 0.81 | Marcus Zamora et al. 2015 |
| *Stephos longipes* | 55 | 168 | 0 | -68.567 | 0.81 | Marcus Zamora et al. 2015 |
| *Stephos longipes* | 51 | 192 | 0 | -68.567 | 0.81 | Marcus Zamora et al. 2015 |
| *Stephos longipes* | 42 | 216 | 0 | -68.567 | 0.81 | Marcus Zamora et al. 2015 |
| *Stephos longipes* | 35 | 240 | 0 | -68.567 | 0.81 | Marcus Zamora et al. 2015 |
| *Stephos longipes* | 28 | 288 | 0 | -68.567 | 0.81 | Marcus Zamora et al. 2015 |
| *Stephos longipes* | 25 | 312 | 0 | -68.567 | 0.81 | Marcus Zamora et al. 2015 |
| *Stephos longipes* | 22 | 336 | 0 | -68.567 | 0.81 | Marcus Zamora et al. 2015 |
| *Tigriopus angulatus* | 1560 | 168 | 6 | -54.638 | NA | Holan et al. 2016 |
| *Tigriopus angulatus* | 892 | 240 | 6 | -54.638 | NA | Holan et al. 2016 |
| *Tigriopus japonicus* | 54 | 96 | 4 | 22.209 | 0.94 | Bao et al. 2008 |
| *Tigriopus japonicus* | 74 | 96 | 10 | 22.209 | 0.94 | Bao et al. 2008 |
| *Tigriopus japonicus* | 20000 | 96 | 4 | 22.209 | 0.94 | Li et al. 2014 |

Table 4. Model summary of the Cox hazard model for the first four exposure days (n = 110, number of events = 29) “concentration” and “species” as additive effects. *C finmarchicus* is used as the reference for the hazard ratio (exp(coef)).

|  | **coef** | **se(coef)** | **z** | **Pr(>\|z\|)** | |  |
| --- | --- | --- | --- | --- | --- | --- |
| Concentration | 0.018253 | 0.002286 | 7.983 | 1.42E-15 | |  |
| C*. glacialis* | -1.481746 | 0.658394 | -2.251 | 0.0244 | |  |
| *C. hyperboreus* | -0.197468 | 0.585077 | -0.338 | 0.7357 | |  |
|  |  |  |  |  | |  |
|  | **Hazard ratio exp(coef)** | **exp(-coef)** | **lower .95** | | **upper .95** | |
| Concentration | 1.0184 | 0.9819 | 1.01387 | | 1.023 | |
| *C. glacialis* | 0.2272 | 4.4006 | 0.06253 | | 0.8259 | |
| *C. hyperboreus* | 0.8208 | 1.2183 | 0.26075 | | 2.5838 | |
|  |  |  |  | |  | |
|  |  | df | p-value | |  | |
| Concordance | 0.974 |  |  | |  | |
| Likelihood ratio test | 118.4 | 3 | <=2e-16 | |  | |
| Wald test | 67.46 | 3 | 1e-14 | |  | |
| Score (logrank) test | 213.4 | 3 | <=2e-16 | |  | |

Table 5 Summary table of all linear mixed effect models testing the influence of environmental variables on reported LC50 (mg L^-1^). In all models, the number of observations is 48, with eight species specified in the random factor.

| **Exposure duration** | **log(LC50) ~ Hemisphere + log exposure duration + (1 \| species)** | | | |
| --- | --- | --- | --- | --- |
| Random effects | Groups | Name | Variance | Std.Dev. |
|  | Species | (Intercept) | 0.2801 | 0.5292 |
|  | Residual |  | 0.7831 | 0.8849 |
| Fixed effects |  | Estimate | Std.Error | t-value |
|  | (Intercept) | 13.9734 | 1.9228 | 7.267 |
|  | Hemisphere | -1.9102 | 0.3758 | -5.083 |
|  | (log) duration (hours) | 13.9734 | 1.9228 | 7.267 |
|  |  |  |  |  |
| **Latitude/Hemisphere** | **log(LC50) ~ log exposure duration + (1 \| species)** | | | |
|  |  |  |  |  |
| Random effects | Groups | Name | Variance | Std.Dev. |
|  | Species | (Intercept) | 0.1101 | 0.3319 |
|  | Residual |  | 0.7778 | 0.882 |
|  |  |  |  |  |
| Fixed effects |  | Estimate | Std Error | t-value |
|  | (Intercept) | 13.2934 | 1.9357 | 6.867 |
|  | Hemisphere | -1.053 | 0.4686 | -2.247 |
|  | (log) duration (hours) | -1.6392 | 0.4035 | -4.062 |
|  |  |  |  |  |
| Taxonomic Order | log(LC50) ~ Order + log exposure duration + (1 \| species) | | | |
| Random effects | Groups | Name | Variance | Std.Dev. |
|  | Species | (Intercept) | 0.5679 | 0.7536 |
|  | Residual |  | 0.768 | 0.8764 |
|  |  |  |  |  |
| Fixed effects | Estimate | Estimate | Std.Error | t-value |
|  | Intercept (Calanoida) | 13.26533 | 2.02421 | 6.553 |
|  | Order Cyclopoida | 0.07368 | 0.89947 | 0.082 |
|  | Order Harpacticoida | 0.10348 | 0.72124 | 0.143 |
|  | (log) duration (hours) | -1.76987 | 0.39252 | -4.509 |
|  |  |  |  |  |
| Temperature | log(LC50) ~ Temperature + log exposure duration + (1 \| species) | | | |
| Random effects | Groups | Name | Variance | Std.Dev. |
|  | Species | (Intercept) | 0.3689 | 0.6074 |
|  | Residual |  | 0.7808 | 0.8836 |
|  |  |  |  |  |
| Fixed effects | Estimate | Estimate | Std.Error | t-value |
|  | Intercept | 13.787712 | 2.062218 | 6.686 |
|  | Temperature | -0.006423 | 0.062383 | -0.103 |
|  | (log) duration (hours) | -1.866786 | 0.393695 | -4.742 |
|  |  |  |  |  |
| Adult size | log(LC50) ~ Adult size + log exposure duration + (1 \| species) | | | |
| Random effects | Groups | Name | Variance | Std.Dev. |
|  | Species | (Intercept) | 0.3412 | 0.5841 |
|  | Residual |  | 0.7867 | 0.887 |
|  |  |  |  |  |
| Fixed effects | Estimate | Estimate | Std.Error | t-value |
|  | Intercept | 13.75865 | 1.98065 | 6.947 |
|  | adultsize | 0.01551 | 0.14308 | 0.108 |
|  | log) duration (hours) | -1.87085 | 0.3817 | -4.901 |

## Supplement figures


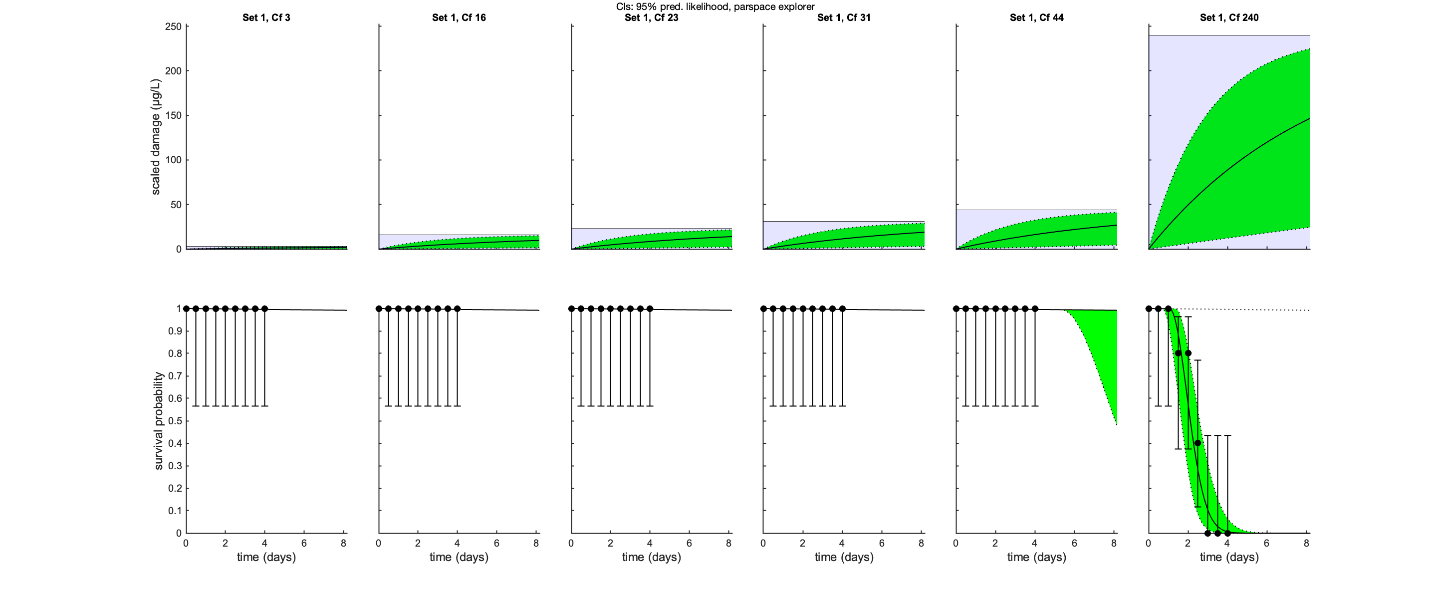


Figure 1 GUTS SD modeled damage and survival fit of *C finmarchicus* from model M2.


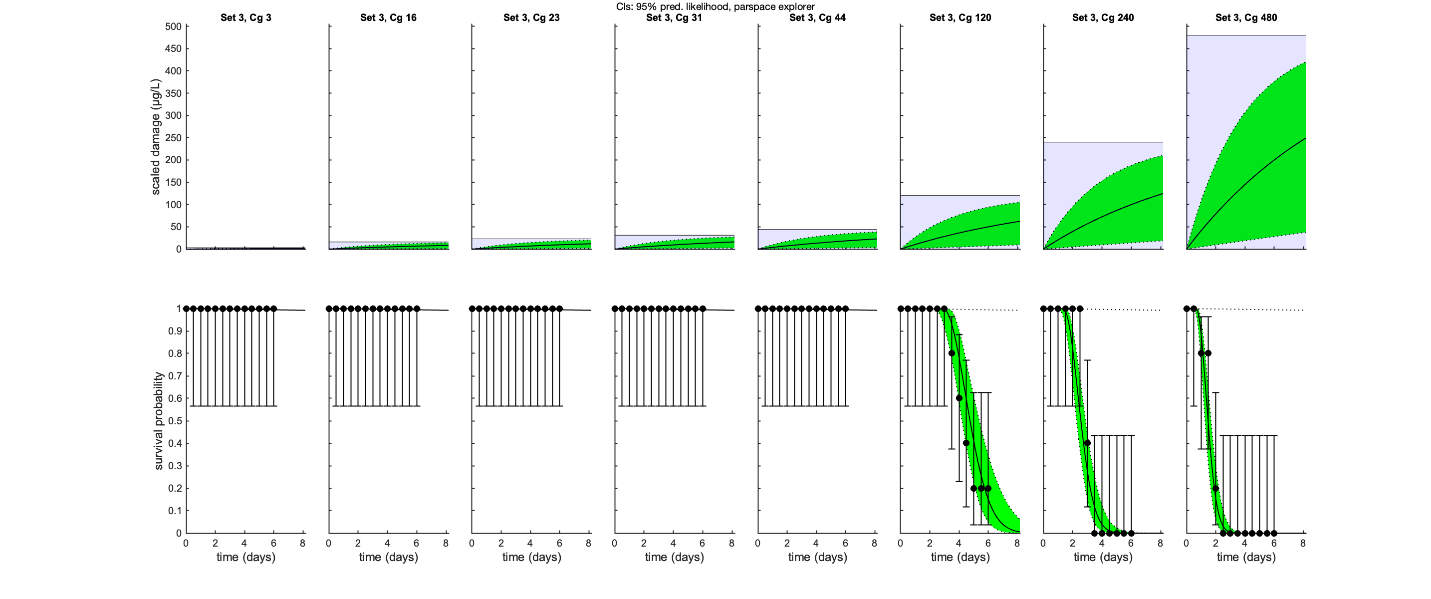


Figure 2 GUTS SD modeled damage and survival fit of *C glacialis* from model M2.


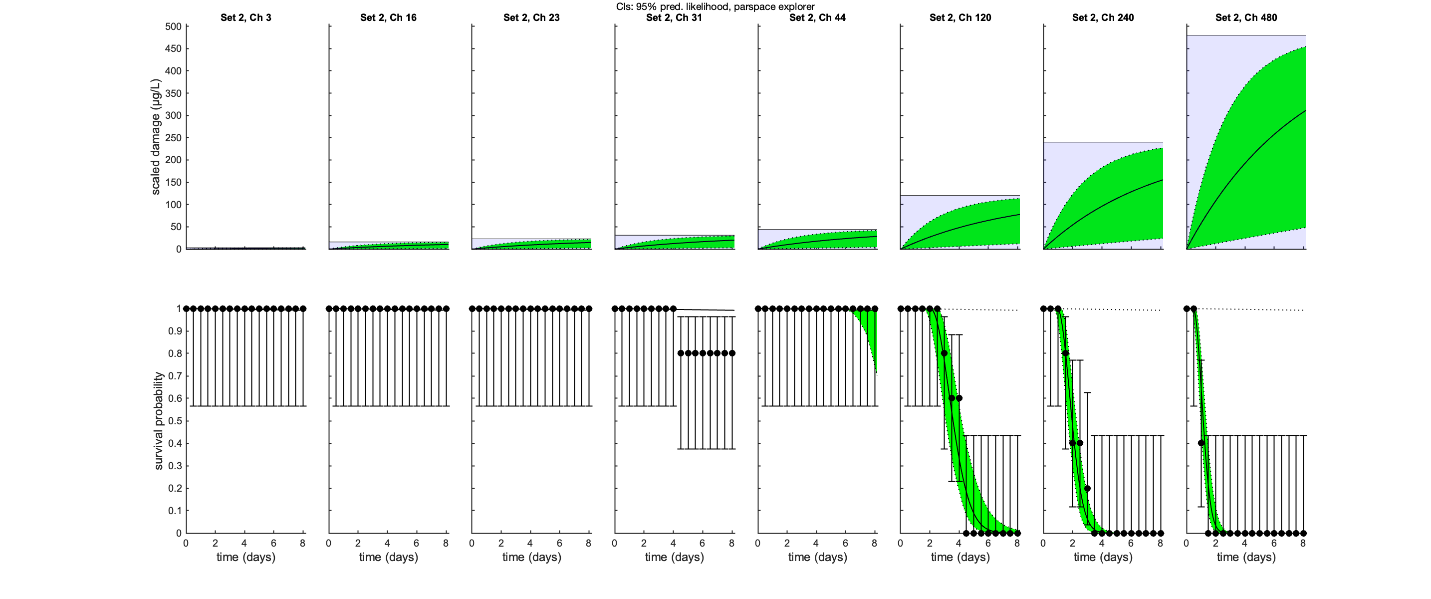


Figure 3 GUTS SD modelled damage and survival fit of *C. hyperboreus* from model M2.


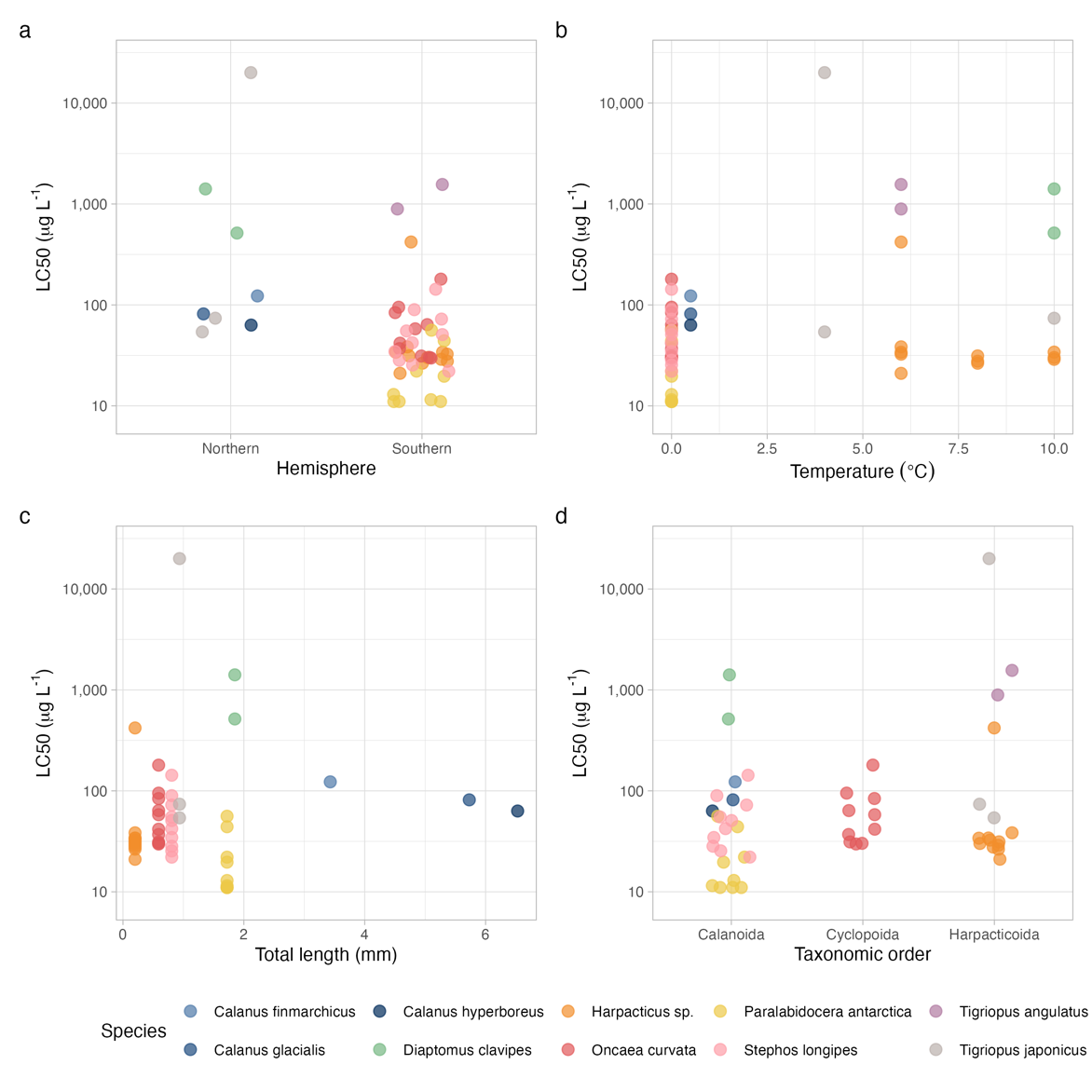


Figure 4 Relationships of the estimated LC50s from this study (Model M1) in the context of published copepod LC50s of copepods done at low temperatures with a) hemisphere, b) temperature, c) total body length and d) taxonomic order. Grey lines indicate model prediction for the respective explanatory variable when significant. Note that the y-axis is on a logarithmic scale.
